# Hydrometallurgical process development to recycle valuable metals from spent SCR deNO_X_ catalyst

**DOI:** 10.1038/s41598-021-01726-0

**Published:** 2021-11-11

**Authors:** Jong Hyuk Jeon, Ana Belen Cueva Sola, Jin-Young Lee, Rajesh Kumar Jyothi

**Affiliations:** 1 Convergence Research Center for Development of Mineral Resources (DMR), Korea Institute of Geoscience and Mineral Resources (KIGAM), Daejeon, 34132 Korea; 2 Department of Resources Recycling, Korea University of Science and Technology (UST), Daejeon, 34113 Korea

**Keywords:** Environmental chemistry, Environmental impact, Environmental chemistry

## Abstract

Spent catalyst, containing vanadium and tungsten oxide in a TiO_2_ glass fiber matrix, pose a risk of environmental contamination due to the high toxicity of its metal oxides if leached into the soil when disposed in landfills. Due to the increasing demand of metals and the continuous depletion of primary resources there is an growing necessity for recycling and reprocessing of spent catalysts and other secondary metal sources for environmental and economical reasons. Study of spent SCR catalyst soda roasting process with dissolved NaOH compared with the usual NaOH dry roasting and its influence in the subsequent water leaching. After optimization, the ideal parameters are roasting using a 0.4 ratio of NaOH/spent SCR catalyst in solution for 2 h at 973 K and de-ionized water leaching for 30 min, at 298 K with a pulp density of 30%. The research results show an important reduction of the roasting temperature and leaching time during the processing of spent SCR catalyst obtaining a 95.4% W and 80.2% V leaching efficiency liquor. Silicon compounds are one of the main impurities leached alongside the valuable metals and in this work, the silicon compounds leached are reduced significantly with the aim of avoiding the de-silication post-processing of the leach liquor. The main advantage of the proposed process is the increase of the leaching efficiency of vanadium and tungsten with a minimization of silicon impurities in a shorter time regardless of the leaching temperature.

## Introduction

From the 2000s (new millennium) the increasing concerns about environmental pollution caused by nitrogen oxides has directly influenced a higher production and demand for selective catalytic reduction (SCR) catalysts for the minimization of the NOx discharged from a variety of sources (stationary and mobile) worldwide^1–3^. Thus, the amount of waste from catalysts generated by increasing the amount of SCR catalysts produced is also increasing^4,5^. Until now, spent SCR catalysts that have been deactivated by poisoning have been regenerated and reused until the activity is reduced, when most of them are disposed in landfills with designated waste based on each country’s regulations^6–8^. However, the composition of the spent catalyst possess another environmental threat when it is disposed or buried due to the accumulation and leaching of V_2_O_5_, a highly toxic compound alongside other heavy metals. In addition, the deactivated catalyst cannot be directly reprocessed as a new catalyst carrier because of the high content of As, Na, Ca, Fe and K and its vulnerability to chemical toxicity^9–11^. One of the most common and effective catalyst are SCR catalysts consisting of V_2_O_5_–WO_3_/TiO_2_ which, generally have very high Ti, W and V content, consequently it can be an economic and environmental feasible option to recover and reuse them. Ti, W, and V remanufactured after recovery can be used as raw materials for new catalysts or as raw materials in other industries^4,12,13^. Accordingly, technology development studies are actively being conducted to recover and remanufacture raw materials from spent catalysts^14^. A variety of hydrometallurgical methods for the recycle of titanium, tungsten and vanadium from spent SCR catalysts are widely known, and they consist in the preparation of a leachate by soda-roasting^15^, pressurized^16^ or acid and alkali leaching, and then purified to obtain the valuable metals in the solution^17^.

Wu et al. (2016) studied the reprocessing of tungsten from spent SCR catalyst (honeycomb type) by alkali leaching-ion exchange method. The spent catalyst samples of 74 μm particle size were leached at a high liquid ratio of 3% and a reaction temperature of 70 °C for 30 min, respectively, and 91 wt% and 87 wt% of W and V were leached, respectively. The leachate was adsorbed using a strong base anion exchange resin (Amberlite IRA900), and the divalent WO_4_^-^ was selectively separated under high pH conditions^18^. Choi et al. (2019, 2018a) performed soda-roasting experiments with Na_2_CO_3_ to react with the spent SCR catalysts. The amount of Na_2_CO_3_ was 10 equivalent and the spent SCR catalyst with a particle size of less than 106 μm was roasted at 1070 K for 120 min. In this study, it was found that the increase of the amount of tungsten leached was related to the inhibition of CaWO_4_ production due to the increase of Na_2_CO_3_ addition along with the rate of TiO_2_ anatase changing phases to rutile. In contrast, the amount of vanadium leached was not influenced by the amount of sodium carbonate, and the results of the experiment showed an approximate constant rate of about 40%. This was attributed to the production of calcium vanadium acid when reacting with CaO present in the raw material, and the leaching ability of V was obtained analyzing the amount of calcium in the raw material^2,10,15^. Wu et al. (2018) examined the selective leaching and reaction mechanisms of V and Fe using oxalic acid. The experiment was performed for 180 min under the condition of a reaction temperature of 90 °C, a high liquid ratio of 20 mL/g, and the particle size of 75 μm using 1.0 mol/L concentration of oxalic acid, the results showed a 84% of V and 96% of Fe leached. The soluble cations VO_2_^+^ and Fe^3^^+^ were reduced through the dissolution and complexation process in the leaching reaction. V and Fe were found to yield a high leaching when they were present in certain forms of VOC_2_O_4_ and Fe(C_2_O_4_)_2_ at 0.33 pH, which showed that the oxidation reduction reaction resulted in the destruction of the dissolution and complexing equilibrium for VO^2^^+^, VO^+^ and Fe^3^^+^. In the case of tungsten and titanium, only the dissolution and complexation reaction occurred and the leaching efficiency was hindered by solubility^19^.

However, in previous studies, if a large amount of SiO_2_ is leached together with vanadium and tungsten, there is a competitive leaching process where vanadium and tungsten leaching could be inhibited by the presence of silicon. In addition, after leaching for separation of the title metals by ion-exchange or solvent extraction, silicon must be removed adding an extra step to the recovery and purification process^15,20–22^.

In this study, we performed a hydrometallurgical process through soda roasting and water leaching for the recovery of vanadium (V) and tungsten (W) from spent SCR deNO_x_ catalysts. The leaching efficiency of vanadium, tungsten and the silicon impurity was compared based on the premise that the phase of the roasting agent, either NaOH solid or NaOH solution, affects the leaching conditions of the title metals and the amount of silicon impurity leached. The optimum leaching condition is derived based on the roasting agent amount, temperature and time. For water leaching the parameters to consider are solid liquid ratio, the reaction temperature, and reaction time.

## Results and discussion

In the soda roasting process, vanadium and tungsten react with NaOH to form the water-soluble compounds NaVO_3_ and Na_2_WO_4_ as shown in reaction 1 and 2. However, in the spent SCR catalysts, in addition to vanadium and tungsten, acting as the main catalytic agents, there is a glass fiber matrix that improves the mechanical properties of the catalyst and its mainly formed of SiO2, therefore Na_2_SiO_3_ is generated (reaction 3) during the roasting process. Being Na_2_SiO_3_ a water soluble compound it is leached alongside V and W which could possibly inhibit or reduce the leaching efficiency of the valuable metals. As a result, the amount of vanadium and tungsten leached decreases due to the presence of silicon soluble compounds. Su et al. (2018) studied the thermodynamics of the three reactions shown below and established that all of the standard free Gibbs energies for the reactions are negative, which indicates spontaneity in the production of the sodium salts, however for temperatures between 0 and 200 °C the most favorable reaction is the vanadium reaction followed by tungsten and silicon^23^.

Therefore, an experiment was performed to reduce the leaching rate of SiO_2_ while maximizing the leaching conditions for vanadium and tungsten.

1 $${\text{V}}_{{2}} {\text{O}}_{{5}} + {\text{ 2NaOH }} \to {\text{ 2NaVO}}_{{3}} + {\text{ H}}_{{2}} {\text{O}}$$

2 $${\text{WO}}_{{3}} + {\text{ 2NaOH }} \to {\text{ Na}}_{{2}} {\text{WO}}_{{4}} + {\text{ H}}_{{2}} {\text{O}}$$

3 $${\text{SiO}}_{{2}} + {\text{ 2NaOH }} \to {\text{ 2Na}}_{{2}} {\text{SiO}}_{{3}} + {\text{ H}}_{{2}} {\text{O}}$$

### NaOH phase and temperature effect

**Figure 1:**
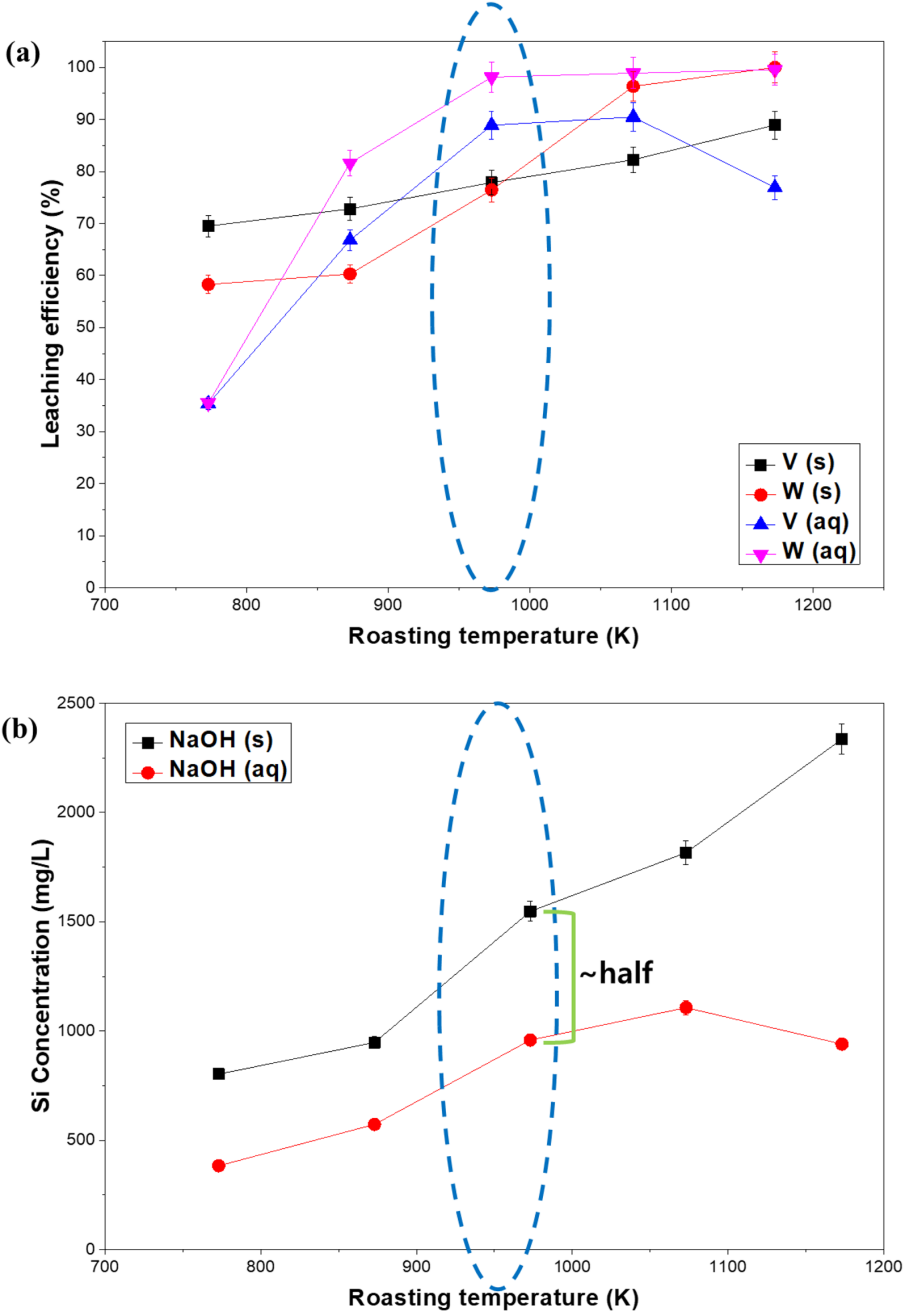
Effects of temperature at the time of roasting process (s = solid, aq = aqueous) in the (**a**) leaching of vanadium and tungsten and (**b**) in the concentration of Si leached.

In the soda roasting process, the reaction temperature is a very important factor in the conversion of V_2_O_5_ and WO_3_ to NaVO_3_ and Na_2_WO_4_ from the feedstock. An experiment was conducted to determine the effect of the phase and reaction temperature of NaOH used as a roasting agent in the amount of vanadium, tungsten and silicon present in the leaching solution. 15 g of NaOH (50% of the feedstock) were added as a solid to the spent SCR catalyst sample and roasted at 773 ~ 1173 K for 2 h while 15 g NaOH were dissolved in a sufficient amount of water and subjected to the same roasting temperatures. After roasting, the residue leached in 100 mL of distilled water at 298 K for 3 h, pulp density = 30%. As shown in Fig. 1a, the leaching efficiency of vanadium and tungsten augmented as the roasting temperature increased. When using dissolved NaOH as a roasting agent, the leaching rate was very low at 773 K, but it increased significantly at 873 K, and it was found to reach the maximum at 973 K. On the other hand, when the roasting agent is solid NaOH, the reaction starts after NaOH melting resulting in a lower leaching efficiency of the title metals. Moreover, dissolved NaOH has superior mass transfer activity with vanadium and tungsten in the spent catalyst and the ionized sodium ions have a higher reaction surface with the spent catalyst particles than NaOH in the molten state. Figure 1a shows that the leaching ability of vanadium and tungsten is maximized when the roasting agent is NaOH solution, while Fig. 1b shows that at the same roasting temperature (973 K) the concentration of silicon leached in the solution is almost half than in the case of NaOH solid used as a roasting agent. The leaching of Si compounds shows that at a higher roasting temperature the amount of Si leached also increases, therefore a lower soda roasting temperature is preferable for the minimization of silicon leaching impurities.

### Effect of sodium hydroxide amount

**Figure 2:**
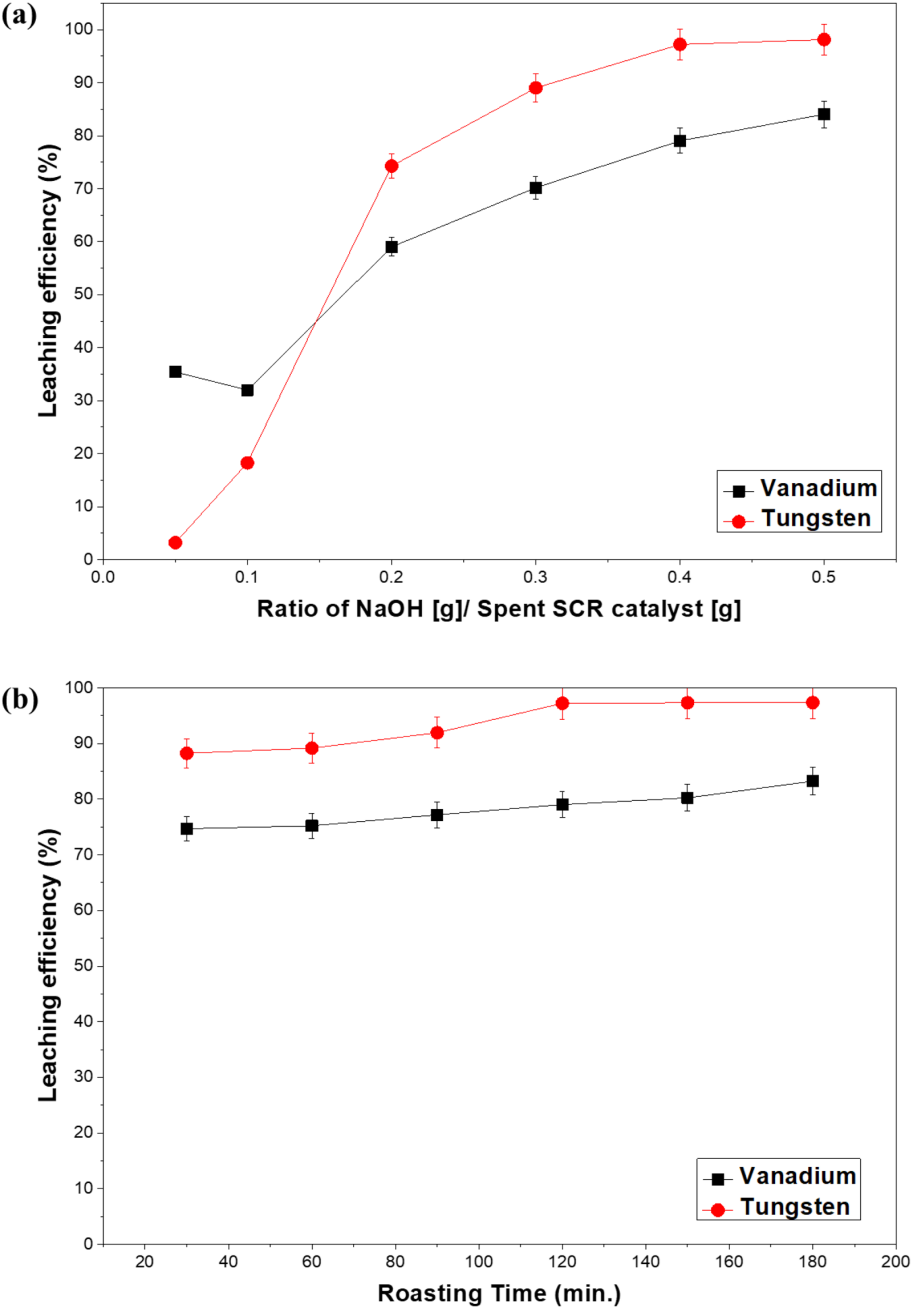
Effect of sodium hydroxide ammount and time for roasting process.

An experiment was conducted by adding the same mass amount of spent SCR catalyst with an aliquot of NaOH (dissolved in enough water) at different ratios ranging from 0.1 to 0.5 of the feedstock to compare the leaching efficiency of vanadium and tungsten. The mixture of NaOH aqueous solution and feedstock was roasted in a muffle furnace for 2 h at 973 K, and after roasting; the sample was added to distilled water and leached at 278 K for 3 h with a pulp density of 30%. The amount of vanadium and tungsten leached according to each experimental condition is shown in Fig. 2a. As the concentration of NaOH augmented, the leaching efficiency of vanadium and tungsten was greater. When the ratio of NaOH/spent SCR catalyst was greater than 0.1, the leaching rate increased significantly, and when the ratio reached 0.4, the leaching efficiency of tungsten reached a maximum, while vanadium leaching increase was insignificant. Therefore, a 0.4 ratio of NaOH/feedstock is considered as optimum for further experiments. In the case of silicon leaching, according to the Le-Chatelier principle, if the amount of NaOH is greater the equilibrium will go towards the formation of products, which in the case include silicon compounds. Thus, a minimization of the amount of NaOH used while maximizing the vanadium and tungsten leached will lead to the minimization of the silicon compounds produced. As analyzed in the previous section, when NaOH solution is used as a roasting agent the amount of Si leached is smaller, therefore at a reduced concentration of NaOH silicon compound production will be smaller than in the case of NaOH solid used as a roasting agent.

### Effects of roasting time

To compare the leaching ability of vanadium and tungsten according to the roasting time, roasting was performed for 30 to 180 min, followed by leaching. As shown in Fig. 2b, leaching efficiency of vanadium increases as the roasting time increases, and the tungsten leaching reaches a maximum after 120 min. Due to the low concentration of vanadium in the original sample and the goal of this investigation being minimizing the amount of water soluble silicon compounds produced during roasting, 120 min is established as the ideal roasting time.

### Effect of pulp density for leaching

**Figure 3:**
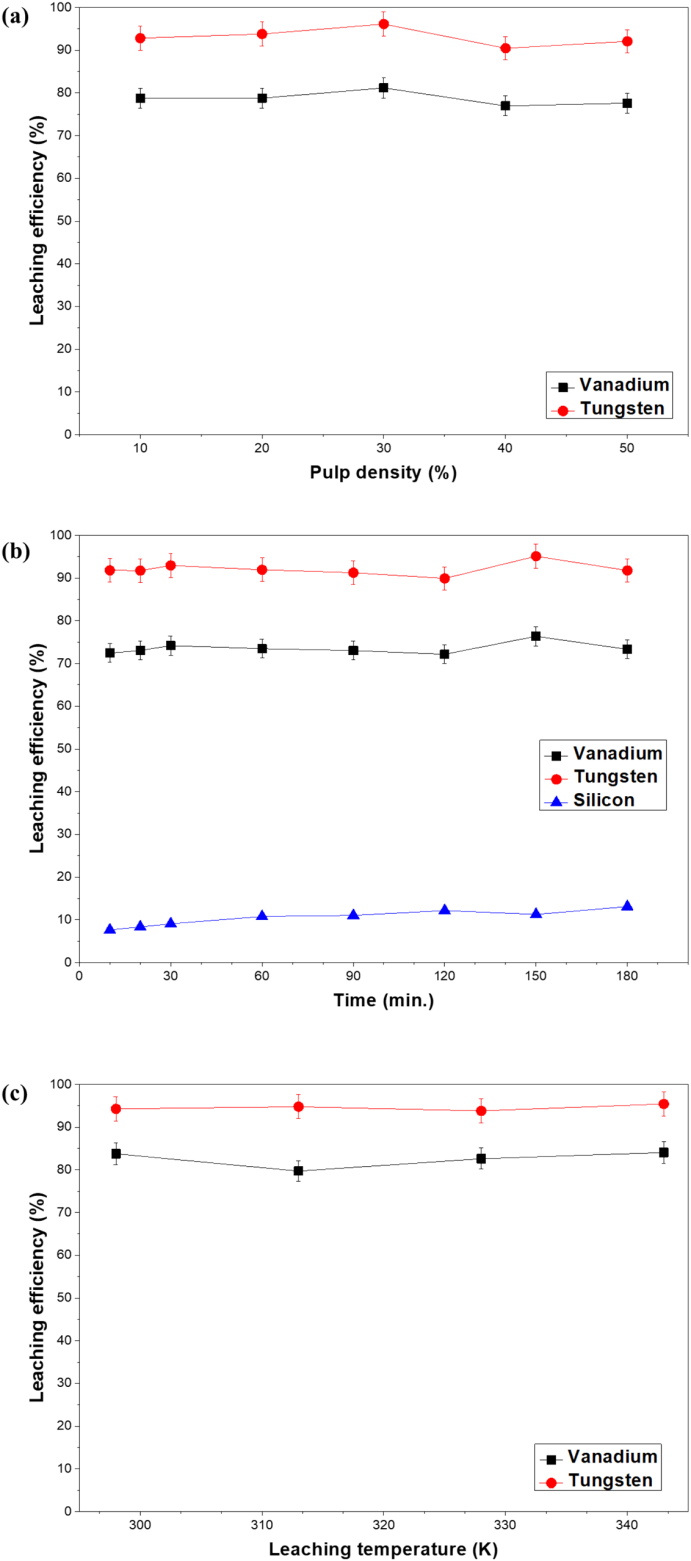
Effect of pulp density (**a**), time (**b**), and temperature (**c**) for leaching of the spent SCR catalyst.

Various experiments were used to examine how the pulp density influence in the leaching at 298 K and 300 rpm for a sufficient time to have complete leaching (120 min). As shown in Fig. 3a, the pulp density does not have a great influence in the leaching efficiency; still, the highest leaching efficiency was at 30% pulp density. While, above 40%, the leaching efficiency decreases. It is expected that if the amount of spent catalyst used is higher, the concentration of vanadium and tungsten leached will be higher too, however, as noticed in Fig. 3a the leaching efficiency does not improve with the increase of the pulp density. Thus, for an ideal mass transfer 30% pulp density was set as the optimum condition.

### Effect of leaching time

**Figure 4:**
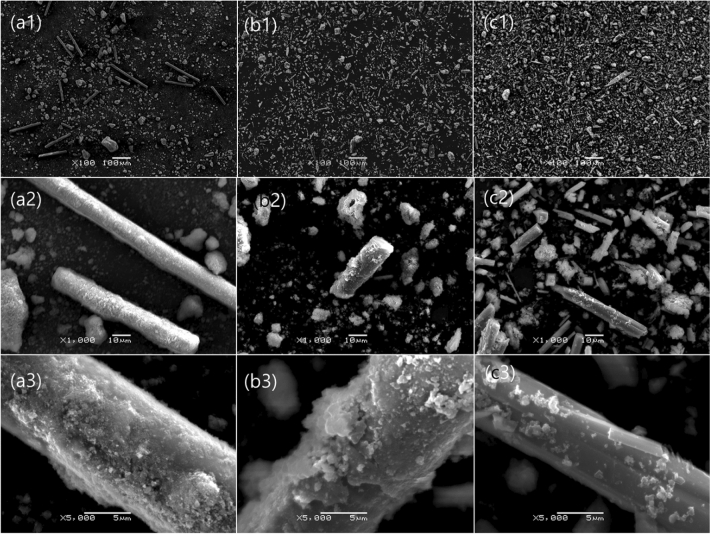
SEM images of (**a**) raw spent SCR catalyst, (**b**) soda roasted spent SCR catalyst and (**c**) residue obtained when water leaching was conducted at 298 K.

The effect of the leaching time from 10 to 180 min in the leaching efficiency of vanadium and tungsten was explored. After soda roasting at 973 K for 120 min, 100 mL of distilled water was used for leaching from 10 to 180 min at 298 K, 300 rpm, and 30% pulp density. As shown in Fig. 3b, the leaching time does not contribute significantly to the leaching efficiency for vanadium and tungsten. Several researchers have studied the ideal conditions for roasting and leaching of spent SCR catalyst, however leaching time has always been long (at least 1 h) to maximize the leaching of vanadium and tungsten^19,20,24,25^. For instance, Moon et al. obtained an almost complete leaching of vanadium and tungsten in a 1 h interval, however they did not consider the amount of silicon leached alongside the title metals^26^. Wu et al. did an alkaline leaching without a preprocessing (soda roasting) of the catalyst which has the disadvantage of a great consumption of alkali in the process and it takes 180 min to maximize the leaching efficiency of the valuable metals^19^. On the other hand, the process studied in this investigation reaches a maximum leaching efficiency of tungsten and vanadium in 30 min with the additional advantage of the minimization of the silicon compounds leached. As observed in Fig. 4c3 the fiber glass matrix (composed mostly of silicon oxide) after leaching keeps its structure, which shows the possibility of more formation of water soluble silicon compounds, thus minimizing the leaching time is imperative to minimize the silicon leached. It is deduced that vanadium and tungsten were converted into easily soluble compounds during the soda roasting process using NaOH aqueous and that facilitated the following leaching.

### Effect of temperature for leaching

In the leaching process, the reaction temperature affects the mass transfer activity of the water-soluble target metals and acts as an important factor to determine the amount of metals leached. The effect of leaching temperature on leaching efficacy of vanadium and tungsten was studied in a range between 298 and 343 K. As shown in Fig. 3c, the leaching efficiency for both title elements does not show a significant correlation with the temperature in the leaching process, this fact leads to the conclusion that from an economic standpoint 298 K will be used as the optimum leaching temperature. Moreover, it has been studied by various authors that as the reaction temperature increases, the activities of substances other than vanadium and tungsten in the spent catalyst increase^2,19,23,27^, therefore a lower temperature (298 K) is preferred to avoid an increase in the leaching of silicon or any other impurity.

### Morphology of the samples at different metal recovery stages

SEM–EDS images were taken for the raw spent SCR catalyst, after roasting and after leaching to compare the morphology of the residues at different stages. Figure 4 shows the SEM images at different magnifications for the original spent SCR catalyst, the spent SCR catalyst roasted with dissolved NaOH and the residue after leaching. Figure 4a1,b1,c1 show the spent catalyst sample before roasting, after roasting and after leaching at low magnification, respectively. It can be observed that in the case of the raw spent catalyst (a1) there is a clear presence of fiber glass rods (silica, alumina and calcium oxide) which are the main concern for this research due to the minimization of silica leaching as a primary research goal. As observed in the posterior EDS figures it is clear that the particles surrounding the glass fiber rods are composed mainly of titanium oxide (anatase form), vanadium and tungsten while the primary structure of the rod is silica. In Fig. 4b1,c1 the rods are not clear in shape at low magnification due to the crushing done with mortar and pestle to the roasted residue before leaching.

When the magnification is increased 1000 times, it can be observed that in Fig. 4a2,b2 there is some agglomeration on the rod type structures while in Fig. 4c) the rod structures show a smooth texture. In the largest magnification Fig. 4a3,b3,c3, it can be observed in the original spent catalyst an agglomeration on top of the fiber glass road which being analyzed by EDS it is attributed to the presence of vanadium and tungsten in the fiber glass matrix of the spent catalyst. Moreover, in Fig. 4b3 there is agglomeration over the rod-type structure, which when analyzed with EDS it shows the formation of a layer of sodium compounds formed on the fiberglass matrix. Finally in Fig. 4c3, a smooth texture is observable characteristic of fiber glass surface, which leads to the deduction that vanadium and tungsten sodium compounds were leached from the surface leaving the fiber glass(composed of calcium, aluminum and silicon) in its original shape.

**Figure 5:**
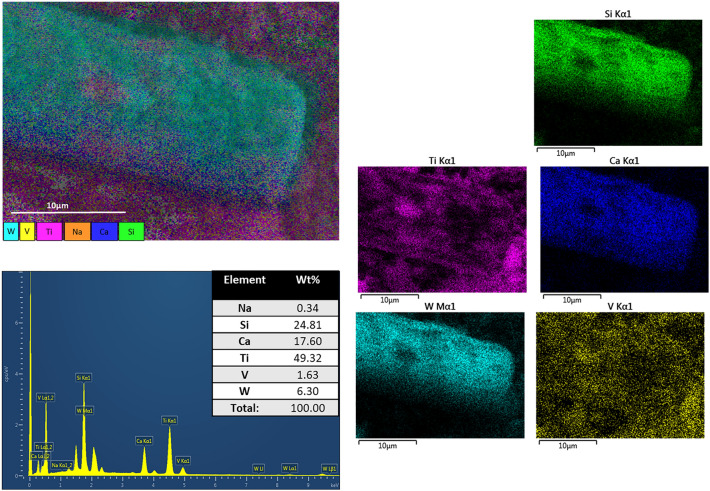
SEM–EDS images and element composition of raw spent SCR catalyst.

In Fig. 5 corresponding to the EDS analysis of the raw spent SCR catalyst it can be appreciated that there is a clear silicon and calcium rod structure in the middle covered mainly in tungsten. Moreover, vanadium and titanium are spread along the sample, the presence of titanium around the rod type structures and spread is in accordance with the main component of SCR catalyst that is TiO_2_ (anatase phase).

**Figure 6:**
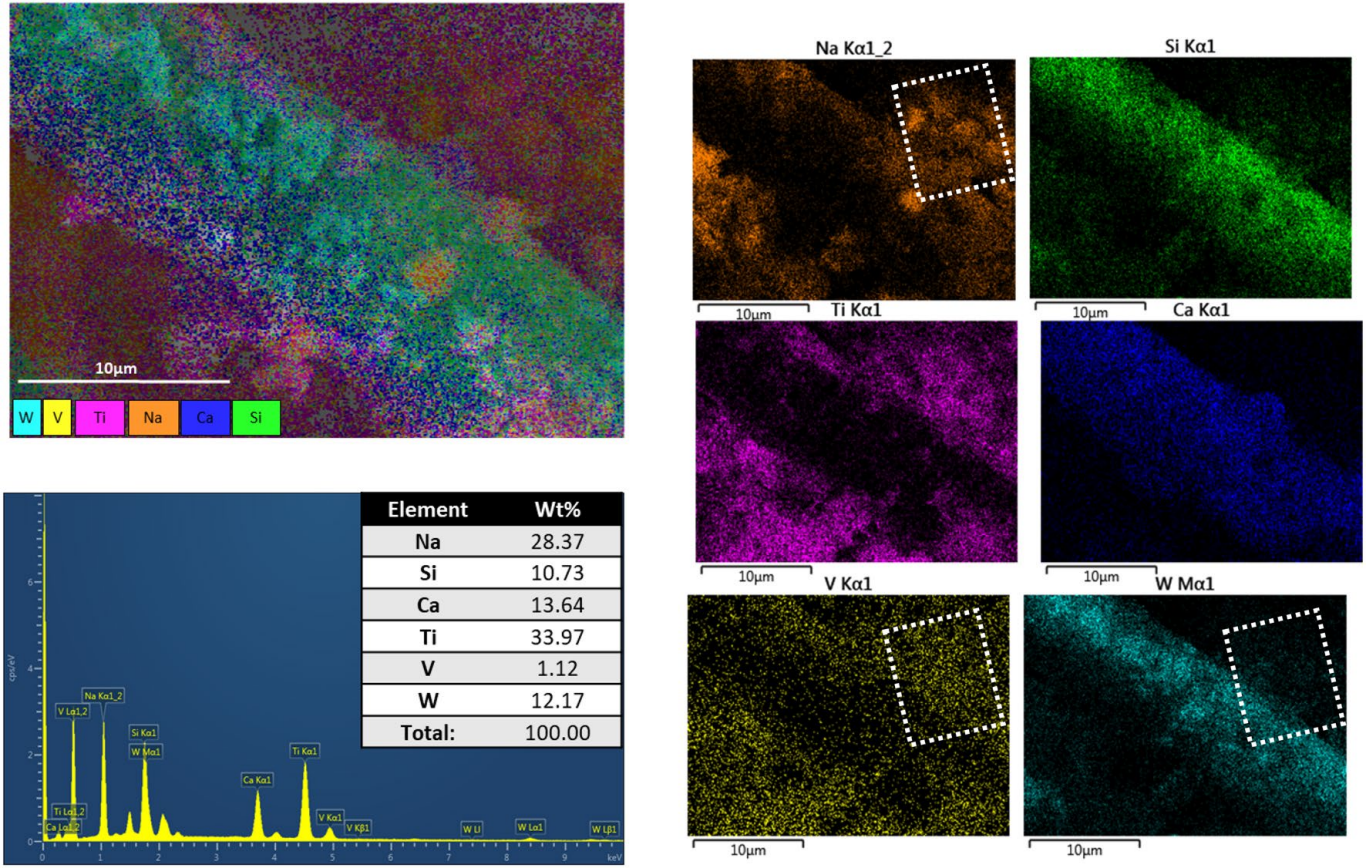
SEM–EDS images and element composition of roasted spent SCR catalyst.

Figure 6 shows the catalyst EDS analysis after roasting, an agglomeration of sodium particles can be observed covering the fiber glass rod which indicate the formation of sodium compounds such as sodium vanadate and tungstate. However, due to the large presence of sodium all over the rod-like structure, it can be deducted that some Na_2_SiO_3_ might have been formed, but due to the uniformity and the high surface area for reaction of NaOH solution with vanadium and tungsten there is a layer formed on top of the fiberglass matrix inhibiting a high leaching of silicon compounds. Moreover, the percentage of sodium in the sample has increased in accordance to the reaction with the metals present.

**Figure 7:**
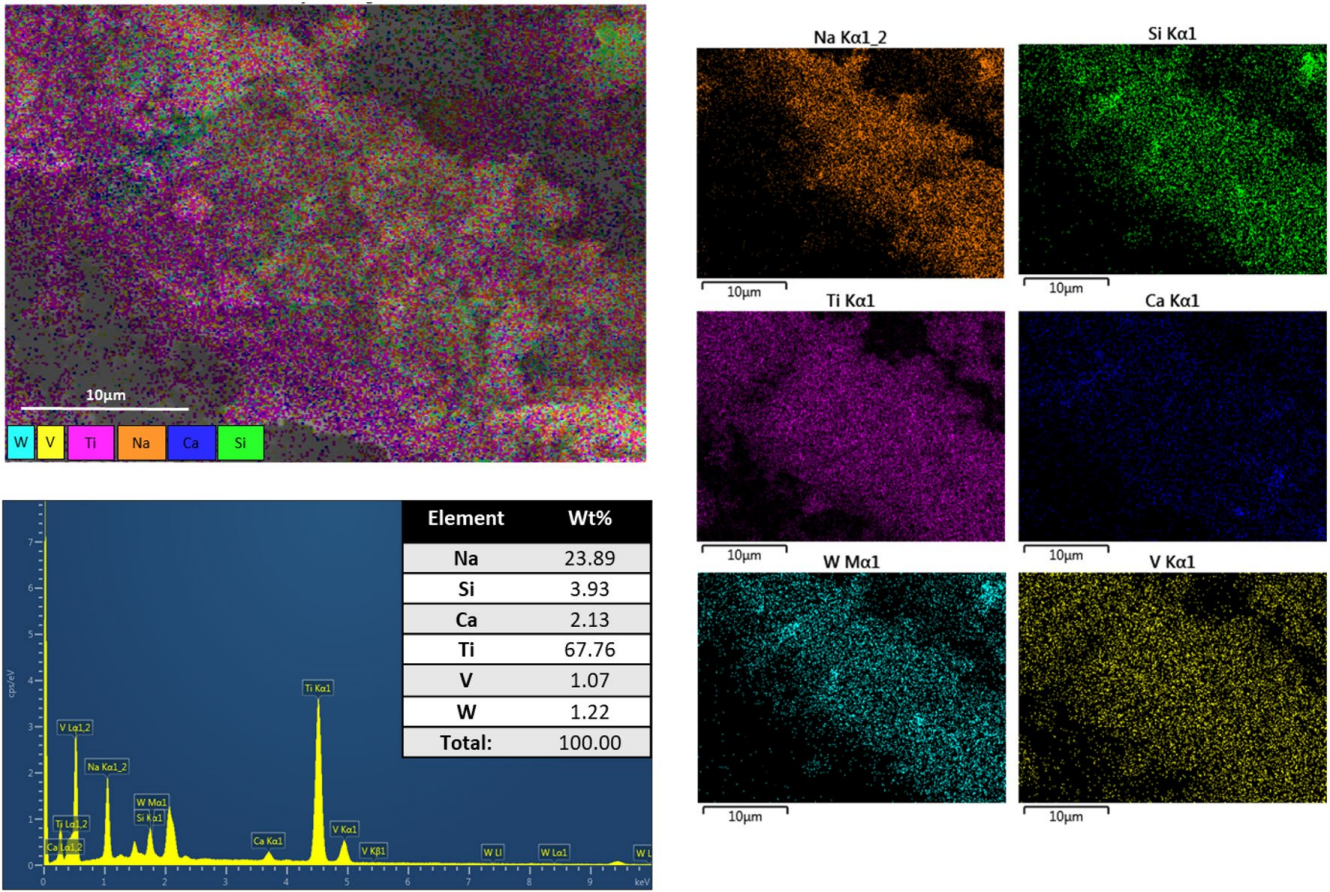
SEM–EDS images and element composition of leached catalyst.

It is observable in the EDS patterns in Fig. 7 that after leaching the density of sodium particles present in the sample decreases, while the percentage of vanadium almost keeps constant (due to the small initial presence of this element) but tungsten decreases significatively. Moreover, the percentage of silicon compounds varies, which is in accordance to the results obtained in the roasting and leaching parameters analyzed previously. In addition, the morphology of the sample keeps the rod-type structure without a great change in shape or dimensions, which leads to the conclusion that a smaller amount of silicon was leached when NaOH solution is used as a roasting agent, in addition some small agglomeration of particles on top of the fiberglass is indicative of vanadium and tungsten not leached during the process.

## Experimental

### Feedstock

**Figure 8:**
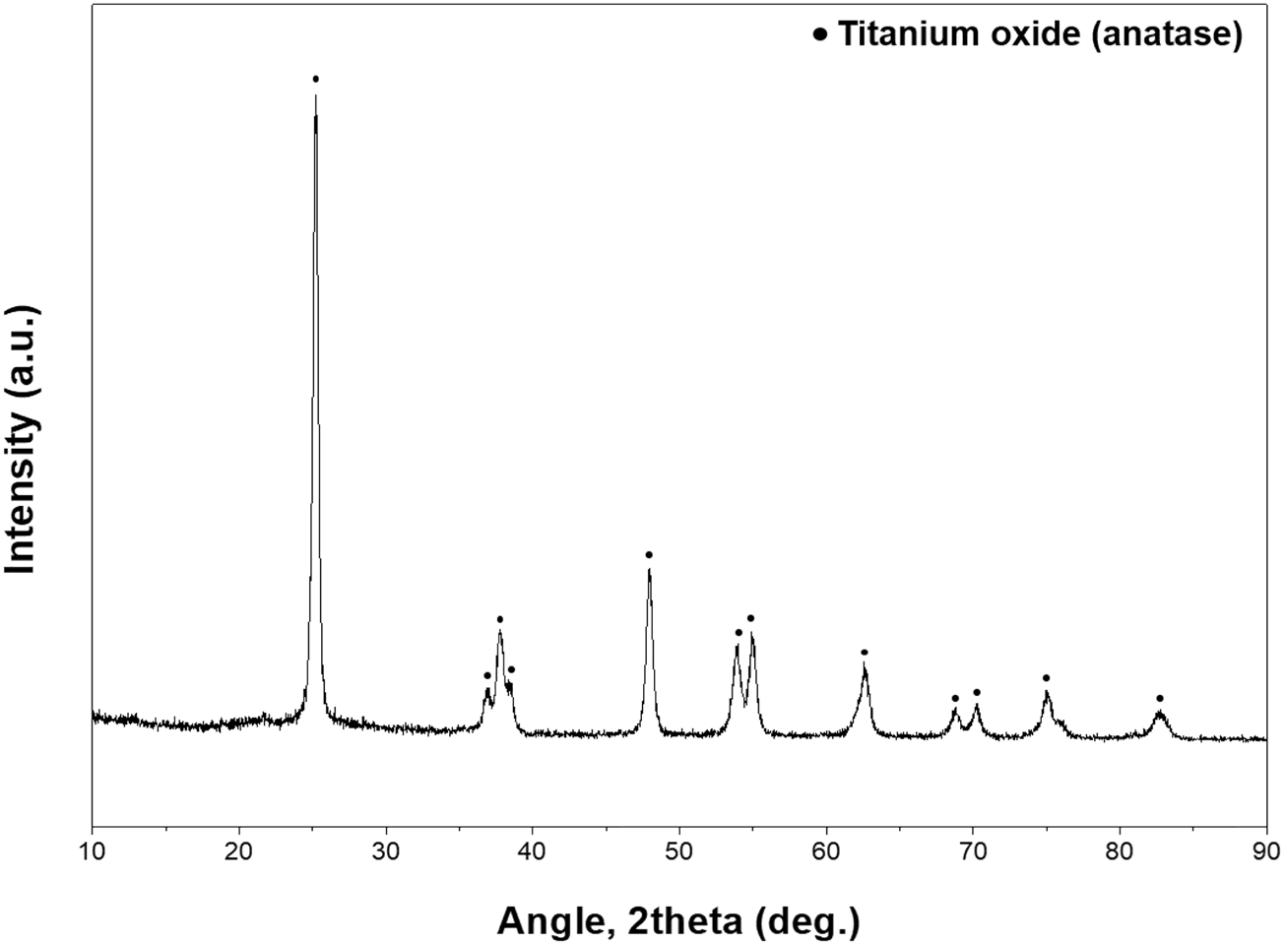
XRD pattern of spent SCR catalyst.

The raw spent SCR catalyst was obtained from the thermoelectric power plant in Samcheonpo, South Korea. The samples were grinded to a size of less than 100 μm, in addition to removing the dust and contaminants on the surface before experiments. The crushed spent catalyst samples were desiccated at 100 °C for a day. The composition of the feedstock used in the experiment is shown in Table 1, and the XRD diagram is shown in Fig. 8.

**Table 1:** Elemental composition of spent SCR catalyst.

Component	TiO_2_	SiO_2_	WO_3_	Al_2_O_3_	CaO	V_2_O_5_	FeO	MgO	MoO_3_	H_2_O
Wt.%	70.9	9.80	7.73	5.57	2.50	1.23	0.77	0.55	0.10	0.85

### Soda roasting and leaching

**Figure 9:**
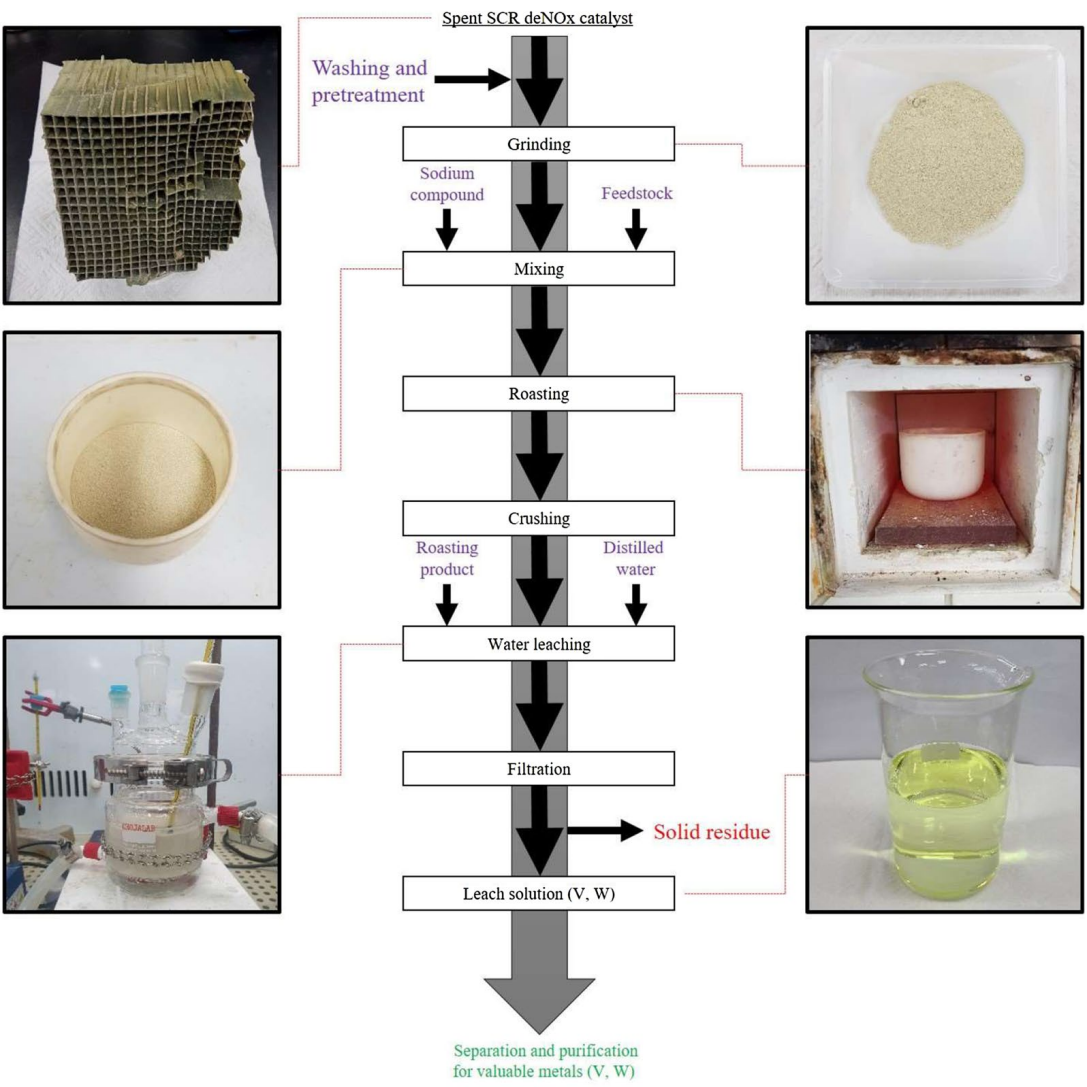
Eco- friendly hydrometallurgical process flowsheet for spent SCR DeNO_x_ catalyst leaching.

Sodium hydroxide (NaOH, supplied by Junsei Chemical, 99% purity, Japan) was used for the soda roasting of the spent SCR deNO_x_ catalyst. NaOH reacts with insoluble V_2_O_5_ and WO_3_ to obtain soluble compounds (NaVO_3_, Na_2_WO_4_) by soda roasting from spent SCR deNO_x_ catalysts. In order to compare the efficiency according to the state (solid or liquid) of NaOH used as a roasting agent, solid NaOH was grinded and mixed with the spent SCR catalyst and placed in an alumina crucible. On the other hand, dissolved NaOH was mixed with the spent SCR catalyst and stabilized for 10 min to wet the solid totally in an alumina crucible in preparation for the roasting process. The amount of NaOH added was 10 to 50% by weight, depending on the weight of the feedstock, and roasted in a muffle furnace without any gas or air inlet for 2 h while varying the temperature conditions. The roasting experiments were performed at temperatures ranging 773 to 1173 K and time variation from 30 to 180 min to understand the effect of the reaction temperature and reaction time. After soda roasting, the cooled sample was placed in a Teflon reactor and leaching experiments were performed on a hot plate modifying different conditions such as pulp density, temperature and reaction time. The overall process for spent SCR catalyst is depicted in Fig. 9.

### Analysis

To obtain the concentration of the title elements (V, W, Si) in the leach liquor an inductively coupled plasma optical emission spectrometer (ICP-OES, Optima 8500, USA) was used. The composition of the raw sample and residues of spent SCR catalyst was analyzed by X-ray diffractometer (XRD, Rigaku, Japan). The scanning electron microscope and energy Dispersive Spectrometer (SEM–EDS, JSM-6380LA, Japan) was used to analyze the surface and composition of samples.

## Conclusions

Due to the environmental risk that spent SCR catalyst pose when discarded in landfills and the presence of valuable metals such as vanadium and tungsten, there is a big necessity for recycling of these secondary resources. During the present research the roasting process of spent SCR catalyst was optimized comparing NaOH dry roasting and aqueous roasting using NaOH solution as a roasting agent. The main goal of the investigation is to maximize the leaching efficiency of both metals while minimizing the silicon compounds leached into the pregnant solution. The optimum conditions for roasting are 0.4 mass ratio of NaOH/ spent SCR catalyst solution for 2 h at 973 K. With a subsequent de-ionized water leaching for 30 min, at 298 K with a pulp density of 30%. At the optimum conditions there was a leaching efficiency of 95.4 ± 3% of tungsten while the vanadium leaching efficiency ranged in 80.2 ± 3%. Research results showed that using NaOH solution as the roasting agent reduced the posterior leaching of silicon compounds and it was observed that at the optimal roasting conditions the amount of silica leached was approximately half than when using dry NaOH as a roasting agent.
